# A versatile, low-cost, and reusable hydroponic system for the cultivation of plants of various species

**DOI:** 10.1007/s44297-026-00084-5

**Published:** 2026-08-06

**Authors:** Jingxiong Zhang, Xin Zhang, Min Qian, Jianqiang Wu

**Affiliations:** 1 Department of Economic Plants and Biotechnology, Yunnan Key Laboratory for Wild Plant Resources, Kunming Institute of Botany, Chinese Academy of Sciences, Kunming, China; 2 State Key Laboratory of Plant Diversity and Specialty Crops, Beijing, China; 3 CAS Center for Excellence in Biotic Interactions, University of Chinese Academy of Sciences, Beijing, China

**Keywords:** Hydroponic system, Crop cultivation, Abiotic stress, Host-parasite interaction, Root physiology

## Abstract

Hydroponic systems are important tools in plant research, as the roots of plants cultivated in hydroponic systems can be easily observed, treated, and sampled. However, the application of many hydroponic systems in research is constrained by species specificity, high costs, and complex assembly requirements. To address these limitations, we developed a versatile, low-cost, and easy-to-assemble hydroponic system capable of supporting the growth of multiple plant species. Here, we demonstrate successful hydroponic cultivation of eight dicotyledonous plant species, including model plants (Arabidopsis and *Nicotiana benthamiana*), horticultural crops (tomato and cucumber), and staple crops (soybean). Importantly, *N. benthamiana*, Arabidopsis, tomato, and soybean can complete their entire life cycles in this hydroponic system and ultimately produce mature seeds. This hydroponic cultivation system has a wide range of applications, particularly for the study of root physiology, host‒parasitic plant interactions, biotic and abiotic stress assays, and root exudates. This system provides a practical platform for plant research in laboratories.

**Supplementary Information:**

The online version contains supplementary material available at 10.1007/s44297-026-00084-5.

## Introduction

With the increasing scarcity of land resources and the growing demand for resource-efficient crop production, soilless cultivation has become an important cultivation method for improving the productivity of grain and vegetable crops [1, 2]. Among these methods, hydroponic culture has emerged as a key tool in plant science research because of its distinct advantages, including simple, clean, and controllable nutrient composition; avoidance of soil-borne pathogen contamination; easy sample collection; and high experimental reproducibility [3, 4]. In particular, hydroponic systems allow precise control of mineral nutrients, including nitrogen, phosphorus, and potassium, and other factors, such as growth regulators and rhizosphere pH [3, 5, 6]. The hydroponic system is also widely used for plant–microbe interactions [7, 8]. In addition, these systems also allow nondestructive observation and quantitative analysis of root development and function [5, 9].

Although soilless systems have wide applications in plant research, many hydroponic systems still have limitations, including narrow species adaptability, insufficient scalability, and limited flexibility [5, 9, 10]. For example, some systems rely on sand, perlite, vermiculite, or other solid substrates to support plants, and many require additional aeration devices, water circulation components, frequent replacement of nutrient solutions, and pH adjustment, all of which significantly increase operational complexity and labor costs [5, 10]. Importantly, small-scale cultivation in laboratories, such as Arabidopsis (*Arabidopsis thaliana*), *Nicotiana benthamiana*, and tomato (*Solanum lycopersicum*), is needed. Many hydroponic systems often suffer from problems such as high equipment complexity, cost, and labor-intensity [5, 9–11]. Furthermore, commercially available hydroponic devices are often optimized for specific crop varieties or require substantial investment, limiting their accessibility and scope of application [1, 2]. There is therefore a need to develop a hydroponic system that is low cost, easy to assemble, and can be used for multiple plant species.

In this study, we designed a simple and low-cost hydroponic system that is suitable for the cultivation of at least 8 dicotyledonous species under laboratory conditions, including the model plants Arabidopsis and *N. benthamiana*, as well as the crops soybean (*Glycine max*), cucumber (*Cucumis sativus*), and tomato. In addition to being used for cultivation, this system can also be used for a wide range of plant biology research, including host–parasitic plant (dodder *Cuscuta australis*) interactions, transient gene expression, grafting, root treatment, and root exudate collection.

## Materials and Methods

### Plant materials and cultivation conditions

Arabidopsis (*Arabidopsis thaliana*, Col-0), soybean (*Glycine max*, cv Huachun), tomato (*Solanum lycopersicum*, cv Micro-Tom for hydroponic culture; Moneymaker for grafting), *Nicotiana benthamiana*, tobacco (*Nicotiana tabacum* cv K326), maize (*Zea mays*, cv A188), cucumber (*Cucumis sativus*, cv Chinese Long), potato (*Solanum tuberosum* cv Phureja S15-65), wheat (*Triticum aestivum* cv No1 Maifeng), rice (*Oryza sativa* cv Nipponbare), *Astragalus membranaceus*, *Cuscuta australis* (laboratory maintained ecotype) [12], and *Phelipanche aegyptiaca* (laboratory maintained ecotype) [13] were used in this study. All the seeds were sterilized with a sodium dichloroisocyanurate solution (0.02 g/ml, containing 0.1% Tween-20) for 10 min, except for those of *Phelipanche aegyptiaca*, which were sterilized for 3 min. After being washed four times with sterile water, the seeds were placed on trays containing perlite and watered with 1/2 modified Hoagland solution (MHS) for germination under controlled conditions (24 °C, 16 h light/8 h dark photoperiod, relative humidity of 60–70%). The 1/2 MHS consisted of 0.5 mM CaCl_2_·2H_2_O, 1 mM MgSO_4_·7H_2_O, 5 mM KNO_3_, 0.5 mM KH_2_PO_4_, 0.01 mM ethylenediaminetetraacetic acid iron(III) sodium salt (C_10_H_12_FeN_2_NaO_8_), 2.86 mg/L H_3_BO_3_, 1.81 mg/L MnCl_2_·4H_2_O, 0.22 mg/L ZnSO_4_·7H_2_O, 0.08 mg/L CuSO_4_·5H_2_O, 0.02 mg/L H_2_MoO_4_·H_2_O, and 0.01 mg/L CoCl_2_·6H_2_O, with a pH of 5.8–6.0.

### Setup of the hydroponic system

The seedlings were cultivated for 15 days on trays and then transplanted into 1-L plastic opaque hydroponic pots, whose lids had been drilled to form a hole (0.5 cm in diameter) in the middle. The pots were filled with 1/2 MHS, which was replaced every two weeks to maintain optimal nutrient availability, and 15-day-old seedlings were transferred into these pots. The 1/2 MHS was always less than 3/4 of the volume to ensure that the base parts of the roots were not submerged in hydroponic solutions.

### Stress treatments

*N. benthamiana* plants were germinated and grown hydroponically on 1/2 MHS medium as described above. Two-month-old *N. benthamiana* plants with uniform growth were selected for stress treatments. The roots were subsequently washed three times with tap water to remove residual nutrient solution, and the plants were randomly divided into four groups and transferred to hydroponic solutions for different treatments: full nutrient, nitrogen deficiency, phosphorus deficiency, and salt stress. The plants in the full nutrient group were supplied with 1/2 MHS. The nitrogen deficiency group received 1/2 MHS, where 5 mM KNO_3_ was replaced with 5 mM KCl, and the phosphorous deficiency group was given 1/2 MHS, where 0.5 mM KH_2_PO_4_ was replaced with 0.5 mM KCl. The plants in the salt stress group were provided with 1/2 MHS supplemented with 200 mM NaCl. During the 30-day stress treatment period, the hydroponic mixture was renewed every 5 days to maintain stable nutrient concentrations and salinity. After 30 days, phenotypic observations were conducted. The fifth leaves from the bottom were harvested for chlorophyll and RNA extraction. The shoots and roots were collected to measure root length and dry mass.

To generate wounded leaves, two-month-old hydroponically and soil-grown *N. benthamiana* plants were subjected to mechanical wounding. The fifth leaf from the bottom was gently damaged by rolling three times along the midveins. To wound the roots, the roots were gently rolled three times. Thirty minutes after wounding, the leaves and roots were harvested, immediately frozen in liquid nitrogen, and stored at −80 °C until subsequent phytohormone analysis.

### Hormone analysis

Jasmonic acid (JA) and jasmonic acid-isoleucine (JA-Ile) contents were quantified using liquid chromatography‒tandem mass spectrometry (LC‒MS/MS) following an established protocol [14].

### Transient expression of RUBY and eGFP

pCAMBIA3301-RUBY and pCAMBIA330-eGFP, which express *RUBY* [15] and *enhanced green fluorescence protein* (eGFP), respectively, and P19 [16] was subsequently transformed into *Agrobacterium tumefaciens* strain GV3101. These strains were subsequently cultured in YEP media to OD_600_ = 0.6–0.8, subsequently centrifuged (5,000 *g*, 10 min) and resuspended in 10 mM MES (2-morpholinoethanesulfonic acid) supplemented with 10 mM MgCl_2_ and 200 μM acetosyringone (pH 5.6; OD_600_ = 1.0). The former two strains were mixed with P19 at a 1:1 ratio, kept in the dark at 25 °C for 2–3 h and then injected into the leaves of 6-week-old *N. benthamiana* plants growing hydroponically and in soil. The expression of RUBY and eGFP were examined after four days of cultivation. RUBY signals were directly photographed by a Canon 5DII digital camera, while eGFP fluorescence was visualized with a UV fluorescent lamp (3415RG, LUYOR) and photographed using a Canon 5DII digital camera equipped with an optical filter.

### Gene expression analysis

Total RNA was extracted from root tissues using TRIzol reagent (Thermo Fisher Scientific) according to the manufacturer’s instructions. First-strand cDNA was synthesized with the Hifair III 1 st Strand cDNA Synthesis Kit (Yeasen Biotech). Quantitative real-time PCR (qRT-PCR) was then performed using iTaq Universal SYBR Green Supermix (Bio-Rad) on a qRT-PCR system (Bio-Rad, CFX Connect). The primer sequences are listed in Table S1, with *NbUBQ* (NbL15g20120) selected as the reference gene.

### Root exudate collection and Phelipanche aegyptiaca germination bioassay

Root exudates were collected from 2-month-old *N. benthamiana* plants subjected to full nutrient treatment or phosphorus deficiency. The plants were subsequently cultured in sterile water for 48 h to allow the release of root exudates into the water. The collected solutions were centrifuged at 12,000 *g* for 10 min at 4 °C, and the supernatants were concentrated to 10 mL using a rotary evaporator (Eppendorf). The concentrated root exudates were filtered through 0.45 μm microporous membrane (Merck Millipore) for sterilization.

For *Phelipanche aegyptiaca* seed germination assays, approximately fifty sterilized seeds were placed on moist filter paper in Petri dishes for two days of imbibition. Next, 100 μL of the concentrated root exudates was added to the filter paper. For the positive control, 100 μL of 10 μM GR24 (a synthetic strigolactone analog, Coolaber) was added. After seven days, the seed germination rates were recorded.

### Dodder infestation

Dodder (*Cuscuta australis*) infestation was performed according to a previously published method [12]. In short, dodder seedlings were used to infest soybean plants. Approximately three weeks later, the vigorously growing branches were excised and used for the infestation of six hydroponically and soil-grown *N. benthamiana* plants. After approximately two weeks, the establishment of parasitism was observed.

### Tomato plant grafting

Approximately 45-day-old *Solanum lycopersicum* cv. Moneymaker plants were used for grafting. Stems were cut obliquely with a sharp blade. Large leaves of scions were removed, and the scions and rootstocks were closely aligned. The graft junction was wrapped with parafilm. The grafted plants were kept in a transparent closed plastic container under weak light for one week, followed by cultivation under normal light.

### Statistical analysis

All the experiments were conducted with at least four biological replicates. The data are presented as the means ± standard errors. Statistical significance was determined using Student’s *t* test or one-way ANOVA followed by Tukey’s HSD test where appropriate. Statistical analyses were performed using R software, and significance levels were set at *p* < 0.05.

## Results

### Construction and nutrient management of a low-cost, and reusable hydroponic system

**Fig. 1:**
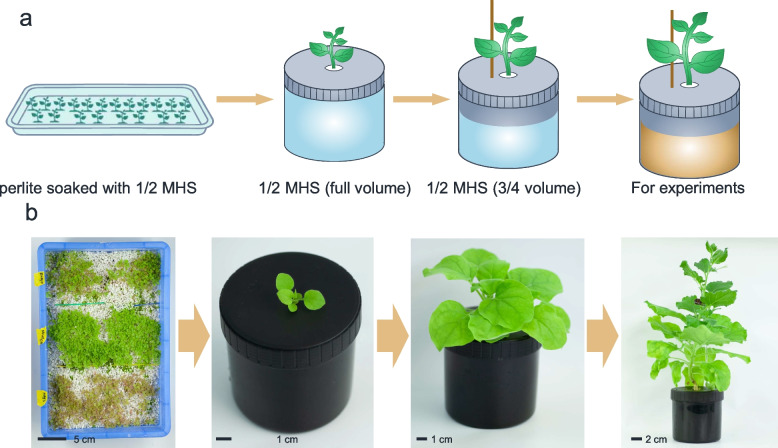
Schematic overview of the hydroponic system and cultivation process for *Nicotiana benthamiana.* **a** Schematic workflow of the hydroponic culture system. Seeds were sown in perlite soaked with 1/2 MHS, and the germinated seedlings were subsequently transplanted into 0.5- or 1-L hydroponic pots filled with 1/2 MHS at the initial stage. For all subsequent medium renewals, the nutrient solution volume was adjusted to approximately 3/4 the volume of the pot. Each pot lid was modified such that a hole was present in the center to hold individual plants and a small hole nearby to accommodate plastic supporting sticks. **b** Representative growth progression of *N. benthamiana* from germination to maturity in the hydroponic system

A simple, low-cost, and reusable hydroponic system was constructed using commercially available 1-L plastic hydroponic pots with matching lids, each of which costs approximately 0.30 US dollars. A hole approximately 0.5 cm in diameter was made in the center of each lid to support the seedling stem and allow the roots to grow into the nutrient solution (Fig. 1a). Additional small holes were made when necessary to insert supporting plastic sticks and clips. The pots and lids can be cleaned, disinfected and reused for subsequent experiments, thereby reducing long-term costs. Unlike soil-based cultivation and more complex hydroponic devices, this hydroponic pot-based system requires no pumps, circulating reservoirs, or specialized devices while allowing controlled nutrient supply and stress treatments.

*Nicotiana benthamiana* has been widely used as a model plant for plant biology research, including for transient and stable transformation and grafting. Thus, we first used *N. benthamiana* to optimize this hydroponic system. The hydroponic solution used in this system was a modified Hoagland solution (MHS). *N. benthamiana* seeds were first germinated on perlite moistened with half-strength MHS (1/2 MHS) (Fig. 1b). When the plants had established robust growth and produced the first true leaves, they were transferred to hydroponic pots. Before transfer, the perlite attached to the roots was carefully removed by dipping the roots in water to minimize root damage (Fig. 1b). The seedlings were then placed into hydroponic pots containing 1/2 MHS, ensuring that the roots were fully submerged in the hydroponic solution during the initial growth stage. To reduce the possible shock induced by the transfer, seedlings were maintained under low-light conditions for the first two days after being transferred to the hydroponic pots. As the root system developed, the solution level was adjusted to approximately 3/4 of the pot volume, allowing adequate aeration of the root system and minimizing the risk of hypoxia caused by complete root submergence in the nonaerated hydroponic solution. The hydroponic solutions were first replaced one month after the plants were transferred to hydroponic pots, after which the solutions were subsequently renewed every two weeks. To prevent plants from tilting during growth, plastic sticks and small clips were inserted through additional holes in each lid to provide mechanical support (Fig. 1b).

### Evaluation of the growth performance and multispecies compatibility of the hydroponic system

To further evaluate the performance and species compatibility of this hydroponic system, seven representative plant species were cultivated under hydroponic and conventional soil conditions. These species included four dicotyledonous plants, namely, cucumber, tomato, soybean, and *N. benthamiana*, and three monocotyledonous plants, namely, rice, wheat, and maize. The plants were first germinated on perlite, after which the plants were subsequently transferred to the hydroponic system and soil culture. Shoot length was measured at 10, 20 and 35 days post-transfer.

**Fig. 2:**
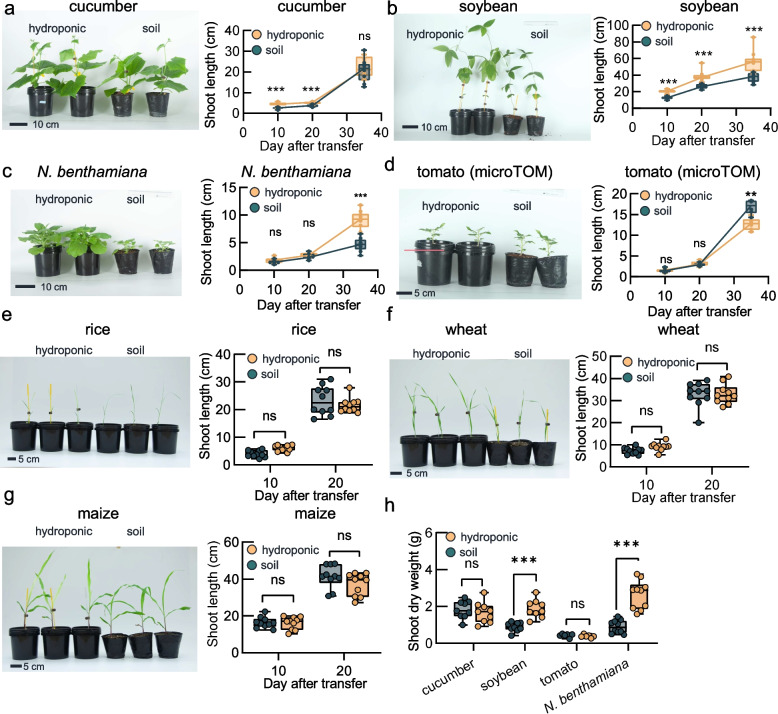
Growth performance of multiple species in the hydroponic system compared with those in soil culture. **a**-**d** Representative shoot phenotypes and shoot lengths of cucumber, tomato, soybean, and *Nicotiana benthamiana* plants grown in the hydroponic system or in soil at days 10, 20, and 35 post-transfer into hydroponic pots (n ≥ 6). **e**–**g** Representative shoot phenotypes and shoot lengths of rice, wheat, and maize grown in the hydroponic system or in soil culture at days 10 and 20 post-transfer into hydroponic pots (n ≥ 6). **h** Shoot dry weights of cucumber, tomato, soybean, and *N. benthamiana* measured on day 35 post-transfer into hydroponic pots. The data are presented as box plots. Boxes indicate the interquartile range from the 25th to 75th percentiles, centerlines indicate medians, and whiskers represent the minimum and maximum values. Statistical significance between the hydroponic and soil treatments was determined using a two-tailed Student’s *t* test; ns, not significant; **p < 0.01; ***p < 0.001

Overall, compared with soil culture, the growth of most species was comparable or improved in the hydroponic system. The shoot lengths of the cucumber and soybean plants were 69% and 63% greater, respectively, under hydroponic conditions than under soil conditions 10 days after cultivation, and this pattern was maintained throughout the cultivation period (Fig. 2a, b, and Table S2). *N. benthamiana* also exhibited enhanced growth in the hydroponic system, with a significant increase in shoot length (almost one-fold) observed on day 35 (Fig. 2c and Table S2). In contrast, compared with hydroponically grown plants, soil-grown tomato plants were 32% taller after 35 days of cultivation, although the shoot dry weights did not differ significantly between these two cultivation systems (Fig. 2d, h, and Table S2). In the monocot species rice, wheat, and maize, no obvious differences in shoot length were observed between the hydroponic and soil culture conditions during the first 20 days after transfer. However, as the cultivation progressed, the plants gradually exhibited chlorosis and poor growth. The repeated optimization of nutrients, including 1/2-, 1-, and 2-strength MHS, was attempted for maize, wheat, and rice; however, compared with soil cultivation, leaf yellowing could not be effectively alleviated, resulting in weak growth under hydroponic conditions. Therefore, the current hydroponic system is only suitable for supporting early-stage monocot growth but requires further improvement for long-term cultivation (Fig. 2e–g and Table S2). Consistent with these growth patterns, the shoot dry weights of hydroponically grown cucumber, soybean, and *N. benthamiana* measured at 35 days post-transfer were significantly greater than those of their soil-grown counterparts (Fig. 2h and Table S2). No significant differences in shoot dry weights were observed for tomato between the two systems. Thus, this hydroponic system supports robust vegetative growth across diverse plant species, with particularly clear growth advantages in several dicotyledonous species.

To further assess the versatility of this hydroponic system, four additional species were tested, namely, Arabidopsis (*Arabidopsis thaliana*), cultivated tobacco (*Nicotiana tabacum*), potato (*Solanum tuberosum*) and the medicinal plant *Astragalus membranaceus*, and these four species all grew in a healthy hydroponic system (Fig. S1).

Importantly, among the 8 dicotyledonous species evaluated in total, four species, *N. benthamiana*, Arabidopsis, soybean, and tomato, successfully completed their life cycles and reached seed maturity in this system. For the remaining species, cultivation was terminated once the plants had reached sufficiently developed vegetative stages, which are adequate for many common experimental applications, including stress-response assays and physiological measurements.

Thus, this low-cost hydroponic system can support the growth of diverse plant species to experimentally relevant developmental stages.

### Application in plant biology research

To further demonstrate the application of this hydroponic cultivation system, we performed multiple comparative experiments between hydroponic and conventional soil culture.

**Fig. 3:**
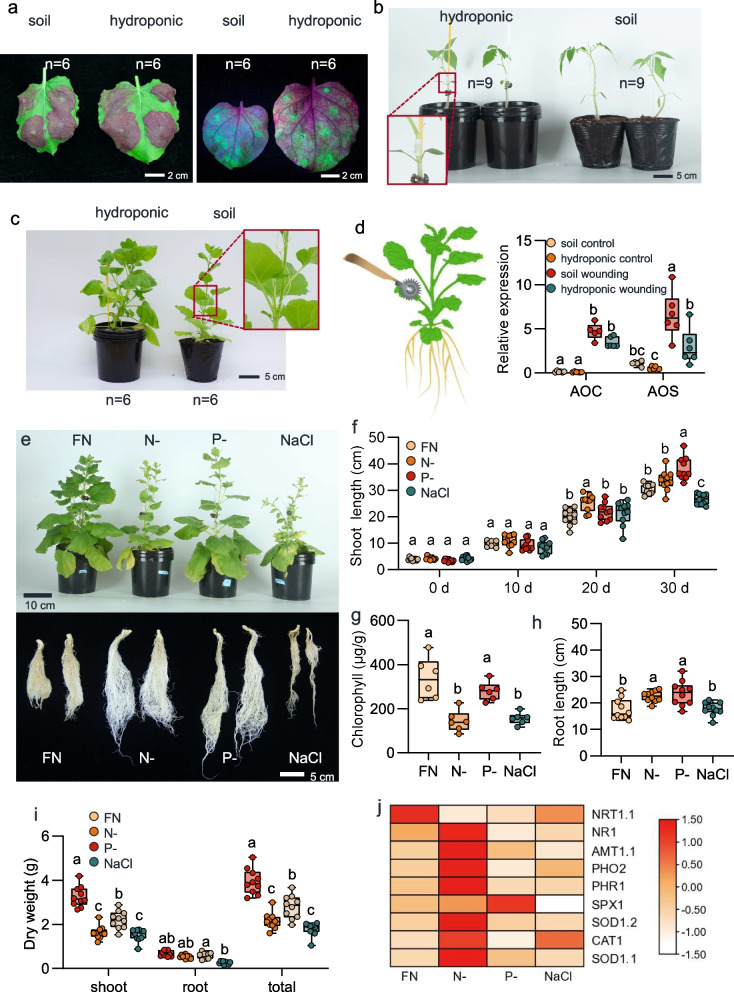
Wide application of the hydroponic system in plant biology research. **a** *Nicotiana benthamiana* leaves transiently expressing the *RUBY* or *eGFP* reporter genes under soil and hydroponic conditions (n = 6). eGFP signals were recorded under UV light. **b** Grafted tomato plants grown under soil and hydroponic conditions. The red box indicates the graft union between the scion and rootstock (n = 9). **c** *Cuscuta australis* parasitizing *N. benthamiana* grown under hydroponic and soil conditions (n = 6). **d** Schematic illustration of mechanical wounding and relative expression levels of the wound-responsive marker genes *AOC* and *AOS* in *N. benthamiana* leaves after mechanical wounding under soil and hydroponic conditions. **e** Photos of the shoots and roots of *N. benthamiana* plants 30 days after different treatments. Full nutrition = FN, nitrogen deficiency = N −, phosphorus deficiency = P −, and salt stress = NaCl. **f** Changes in the shoot lengths of *N. benthamiana* plants under different treatments over 30 days (n ≥ 9). **g-i** Chlorophyll contents (**g**), root lengths (**h**), dry masses of shoots and roots, and total dry masses (**i**) of *N. benthamiana* after 30 days of treatment (n ≥ 6). **j** Heatmap showing the expression patterns of marker genes associated with nitrogen starvation, phosphorus starvation, and salt stress. The relative levels of *NRT1.1*, *NR1*, *AMT1;1*, *PHO2*, *PHR1*, *SPX1*, *SOD1-2*, *CAT1*, and *SOD1-1* were determined by qRT‒PCR (n = 6). The box spans the 25th to 75th percentiles, the line inside the box indicates the median, and the whiskers extend to the minimum and maximum values. Different letters indicate significant differences analyzed by one-way ANOVA (*p* < 0.05)

Transient gene expression assays are widely used in plant research, and *N. benthamiana* serves as an important model. Healthy and vigorous growth is essential for successful transient transformation. The plants cultivated via our hydroponic system grew robustly and achieved high transformation efficiency, as visualized by the RUBY [15] and eGFP fluorescent reporters, which were comparable to those under soil cultivation (Fig. 3a). Grafting is typically applied to explore systemic signaling, nutrient redistribution, and metabolite transport in plants. All nine plants were successfully grafted under each of the two cultivation modes (Fig. 3b). We further investigated whether hydroponically grown *N. benthamiana* can be used for studying interactions between parasitic plants and their hosts. No obvious differences were observed in the parasitization behavior of *Cuscuta australis* on host plants under hydroponic or soil conditions (Fig. 3c).

Root-related experiments, such as sampling, observation, and measurement, are difficult to perform on soil-grown plants, but this hydroponic system allows root observation and convenient treatment/manipulation and sampling of roots and hydroponic solutions. Compared with soil culture, hydroponic culture provides a convenient and reproducible means for the collection of root exudates. To demonstrate the application of this hydroponic system in the collection of root exudates, we collected the root exudates of *N. benthamiana* plants under phosphorus stress and evaluated the root exudate-induced germination rate of the broomrape *Phelipanche aegyptiaca*. Consistent with previous reports [17], the root exudates from phosphorus-starved plants markedly promoted broomrape germination, with a maximum germination rate of 76%, which was similar to that induced by the positive control GR-24 (Fig. S2a and S2b). Next, we compared whether hydroponically cultivated plants respond to mechanical wounding in the leaves and roots of *N. benthamiana*. Both *AOC* (*allene oxide cyclase*) and *AOS* (*allene oxide synthase*), the key genes involved in jasmonic acid biosynthesis [18, 19], were strongly induced following the wounded leaves of hydroponically cultured *N. benthamiana*, although the levels were somewhat lower than those in the soil-cultured plants (Fig. 3d). Wounded roots triggered rapid accumulation of JA and JA-Ile at 30 min, accompanied by upregulation of *AOC* transcripts (Fig. S2c-2f and Table S3).

To demonstrate that this hydroponic system can be easily used for precise control of different abiotic stresses, such as nutrient deficiency, salinity and chemical treatments, we first treated *N. benthamiana* with nitrogen deficiency, phosphorus deficiency, and salt stress, respectively, and monitored the changes in the plants for 30 days. No obvious differences in height were observed among the groups on day 10 (Fig. 3e). Compared with the control plants, the plants in the nitrogen deficiency treatment group were relatively taller on day 20; on day 30, the phosphorus-deficient plants reached a maximum height of 38.2 cm, whereas salt stress severely suppressed growth, with an average height of only 26.0 cm (Fig. 3f and Table S4). Severe leaf yellowing was observed in nitrogen-deficient plants, and necrotic lesions emerged on the leaves under salt treatment (Fig. 3e). Similarly, the chlorophyll contents of *N. benthamiana* leaves markedly decreased under nitrogen and salt stress (57% and 53%, respectively) (Fig. 3g and Table S4). The root length increased under nitrogen and phosphorus starvation but was dramatically inhibited by salt stress (Fig. 3e, 3 h, and Table S4). We then quantified the shoot, root and total dry weight on day 30. Compared with the control treatment, nitrogen deficiency, phosphorus deficiency, and salt stress decreased the average shoot biomass by 50%, 32%, and 53%, respectively (Fig. 3e, 3i, and Table S4). The relative expression of stress marker genes, such as the nitrogen-responsive genes *NRT1.1* (*nitrate transporter 1.1*), *NR* (nitrate reductase), and *AMT1.1* (*ammonium transporter 1.1*)), the phosphorus-related genes *PHO2* (*phosphate 2*), *PHR1* (*phosphate starvation response 1*), and *SPX* (*SPX domain gene*), and the salt tolerance-related genes *SOD1.1* (*superoxide dismutase 1.1*), *SOD1.2* (*superoxide dismutase 1.2*), and *CAT1* (*catalase 1*), was consistent with the stress treatments (Fig. 3j and Table S3).

Taken together, these experiments indicate that this hydroponic system can be used for many areas of plant biology and is especially suitable for root-related research.

## Discussion

Hydroponic cultivation is widely used in plant research because it enables the precise control of nutrient supply, water availability, and chemical treatments while minimizing the heterogeneity commonly associated with soil-based cultivation. These features make hydroponic systems particularly useful for studies involving root growth and development, root exudate, nutrient transport, and stress responses [20–26]. However, many existing hydroponic systems require specialized containers, aeration devices, circulating reservoirs, or frequent maintenance; thus, many systems are both technically complex and relatively costly, limiting their routine use in small-scale laboratories [11, 27–29].

This hydroponic system offers at least three notable advantages. 1) It is inexpensive and reusable. Each pot costs only approximately 0.30 US dollars and can be cleaned for later use, making this system affordable for most laboratories. 2) Easy to use. After they germinate, the seedlings are transferred directly into hydroponic pots, and no other steps are needed. At the early growth stage, the nutrient solution only needs to be replaced after one month of culture. Moreover, opaque pots are light impenetrable, effectively suppressing algal growth in the nutrient solution and minimizing effort in terms of pot cleaning. 3) No need for artificial aeration. All the dicotyledonous plants tested thrived without artificial aeration, which is likely because partial exposure of the basal parts of the roots to air provides sufficient oxygenation to sustain robust root growth. Our experiments demonstrated that this hydroponic system supported robust plant growth in diverse species (Fig. 2). Among the seven species directly compared between hydroponic and soil culture, the shoot growth or biomass accumulation of cucumber, soybean, and *N. benthamiana* improved under hydroponic conditions, whereas those of rice, wheat, and maize generally increased compared with those of the soil-grown plants at early stages. Although this hydroponic system cannot enhance the growth of all species, it provides stable and reproducible growth conditions for many plants. Notably, it is often considered difficult for Arabidopsis to grow hydroponically, but when this system is used, the plants grow well and can even produce seeds (Fig. S1).

Beyond cultivation, this system can be used for various experiments. Like those grown in soil, *N. benthamiana* plants grown hydroponically showed good RUBY activity and eGFP activity during the transient expression assays. The system is also suitable for grafting, root development analysis, root and shoot treatments, shoot and root parasitic plant infestation, and root exudate collection. In particular, the results of the abiotic stress treatment experiments demonstrated that this platform can provide well-controlled stress conditions. In addition, plant–microbe interactions have become a frontier in plant biology research. Further studies are needed to determine whether this hydroponic system is suitable for investigating the interactions between host plants and microorganisms, such as arbuscular mycorrhizal fungi (AMF) and rhizobia [7, 8].

Although the hydroponic cultivation system established in this study can meet the requirements of most seedling-stage experiments, how it can be used for long-term plant cultivation still requires further optimization. Despite our attempt, the current hydroponic system is not suitable for the cultivation of monocots. This may be because the nutrient composition of MHS is not suitable for testing the monocotyledonous plants rice, wheat, and maize. For example, hydroponic solutions containing nitrogen in the form of ammonium or mixed ammonium and nitrate should be tested for monocots. Furthermore, the pH and other elements in different compounds, for example, different iron-containing compounds, should also be tested.

In conclusion, we developed a simple, low-cost, and reusable hydroponic system based on commercially available hydroponic pots and homemade MHS. The system enables robust growth of 8 dicotyledonous plant species and likely many more, and importantly, this hydroponic system can be used for various experiments. Owing to its simplicity, affordability, and broad applicability, this hydroponic system offers a practical tool for laboratories with limited resources and provides a useful platform for experimentation in plant biology.

## Supplementary Information

Supplementary Material 1: Fig. S1 Growth performance of *Arabidopsis thaliana*, *Nicotiana tabacum*, *Solanum tuberosum*, and *Astragalus membranaceus*. a–d Photos of *Arabidopsis thaliana* (a), *Nicotiana tabacum* (tobacco) (b), *Solanum tuberosum* (potato) (c), and *Astragalus membranaceus*(d) cultivated hydroponically. Fig. S2 Applications of the hydroponic system for root-related experiments. a, b Collection of root exudates. *N. benthamiana* plants were subjected to nutrient or phosphorous deficiency, and the root exudates were collected and used for the*Phelipanche aegyptiaca* germination assay. Photograph of germinated *Phelipanche aegyptiaca *seeds (a). Germination rates of *P. aegyptiaca* seeds under different treatments: control (water), root exudates (full nutrient = FN; phosphorus deficiency = P-), and GR24 (n=10) (b). c Schematic of the mechanical wounding treatment of roots. d–f Jasmonic acid (JA) content (d), JA-Ile content (e), and relative expression of *AOC* (f) in roots 30 min after mechanical wounding (n=6). The box spans the 25th to 75th percentiles, the line inside the box indicates the median, and the whiskers extend to the minimum and maximum values. Different letters indicate significant differences analyzed by one-way ANOVA for panel b (*p* < 0.05). Significance levels between treatments were determined by two-tailed Student’s *t *test for panel d, e, and f (***p < 0.001).

Supplementary Material 2: Table S1 Primers used in this study, Table S2 Growth performance of seven plant species in hydroponic and soil culture systems, Table S3 Relative gene expression levels determined by qRT‒PCR, Table S4 Growth performance of *Nicotiana benthamiana* under stress treatments.

## Data Availability

Not applicable.

## Abbreviations

MHS: Modified Hoagland solution
WT: Wild-type
JA: Jasmonic acid
JA-Ile: JA-isoleucine
AOC: Allene oxide cyclase
qRT‒PCR: Quantitative real-time PCR
NRT1.1: Nitrate transporter 1.1
NR: Nitrate reductase
AMT 1.1: Ammonium transporter 1.1
PHO2: Phosphate 2
PHR1: Phosphate starvation response 1
SPX: SPX domain gene
SOD 1.1: Superoxide dismutase 1.1
SOD1.2: Superoxide dismutase 1.2
CAT1: Catalase 1
