# Supplementary material for: A versatile, low-cost, and reusable hydroponic system for the cultivation of plants of various species
Source: Crop Health. 2026 Aug 6;4(1):21. doi: 10.1007/s44297-026-00084-5 (PMC13442767; doi:10.1007/s44297-026-00084-5)
Supplement: Supplementary file 1 [file 44297_2026_84_MOESM1_ESM.pdf]

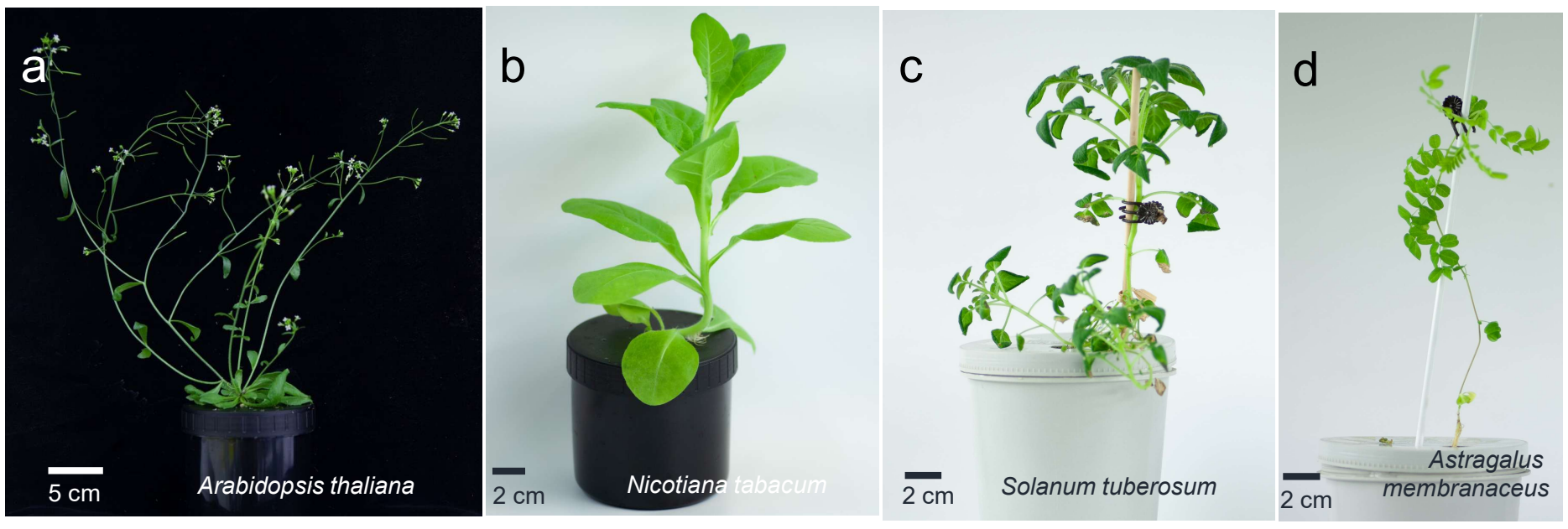

Fig. S1 Growth performance of *Arabidopsis thaliana*, *Nicotiana tabacum*, *Solanum tuberosum*, and *Astragalus membranaceus*.

a–d Photos of *Arabidopsis thaliana* (**a**), *Nicotiana tabacum* (tobacco) (**b**), *Solanum tuberosum* (potato) (**c**), and *Astragalus membranaceus* (**d**) cultivated hydroponically.

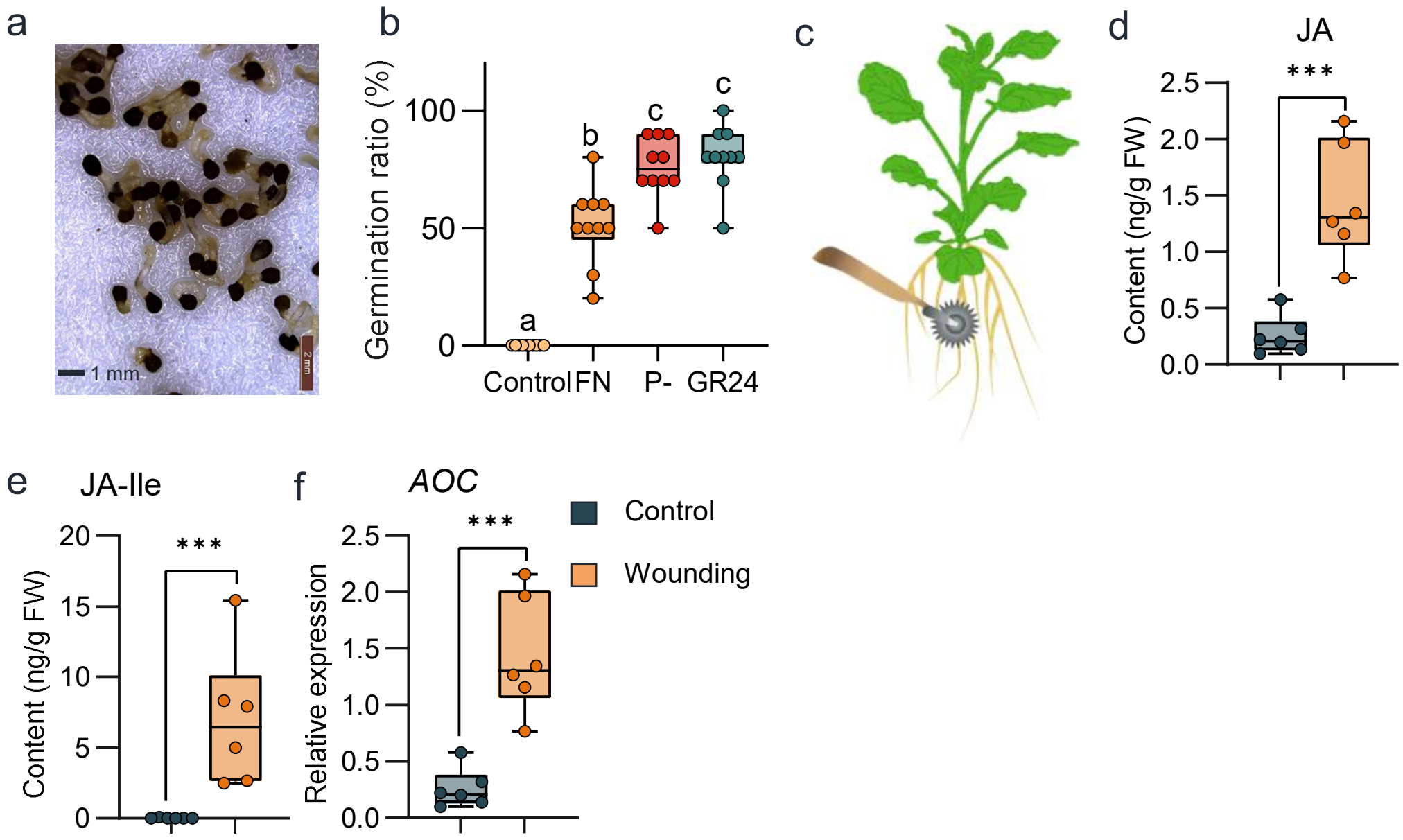

Fig. S2 Applications of the hydroponic system for root-related experiments.

a, b Collection of root exudates. *N. benthamiana* plants were treated with full nutrient or phosphorous deficiency and the root exudates were collected and used for *Phelipanche aegyptiaca* germination assay. Photograph of germinated *Phelipanche aegyptiaca* seeds (**a**). Germination rates of *P. aegyptiaca* seeds under different treatments: control (water), root exudates (full nutrient = FN; phosphorus deficiency = P-), and GR24 (n=10) (**b**). **c** Schematic of mechanical wounding treatment on roots. **d–f** Jasmonic acid (JA) content (**d**), JA-Ile content (**e**), and relative expression of *AOC* (**f**) in roots 30 min after mechanical wounding (n=6). The box spans the 25th to 75th percentiles, the line inside the box indicates the median, and the whiskers extend to the minimum and maximum values. Different letters indicate significant differences analyzed by one-way ANOVA for panel b ($p < 0.05$). Significance levels between treatments were determined by two-tailed Student's t test for panel d, e, and f (***$p < 0.001$).
